# Giant Arcuate Lupus Vulgaris with Rapid Progression: A Case Report

**DOI:** 10.31729/jnma.4453

**Published:** 2019-08-31

**Authors:** Niraj Parajuli, Harendra Kumar Jha, Marcel Franciscus Jonkman

**Affiliations:** 1 Department of Dermatology and Venereology, National Academy of Medical Sciences, Bir Hospital, Kathmandu, Nepal; 2 Chitwan Hospital, Bharatpur, Nepal; 3 Department of Dermatology, University Medical Center, Groningen, Netherlands

**Keywords:** *cutaneous tuberculosis*, *lupus vulgaris*, *Nepal*

## Abstract

Lupus vulgaris is the commonest form of cutaneous tuberculosis. It is a chronic and slowly progressive disease. It can be transmitted either through hematogenous or lymphatic spread but most commonly through contiguous extension. There are many reports on different form of lupus vulgaris but there are only a few reports on large sized lupus vulgaris. Here, we report a case of 75-year-old man with a giant lupus vulgaris rapidly progressing in just two year's duration.

## INTRODUCTION

Lupus vulgaris is the most common form of cutaneous tuberculosis which is chronic and progressive in nature.^1^ This form of cutaneous tuberculosis is found in individual with moderate immunity and a strong tuberculin sensitivity. A characteristic feature of lupus vulgaris is an extremely chronic course with slow but steady growth of the lesions over a period of many years, or even decades.

We present here a case of giant arcuate shaped lupus vulgaris, rapidly progressing over a period of just two years.

## CASE REPORT

A 75 years old male came to dermatology out-patient with a single large atropic, hypopigmented to skin colored plaque on antero-lateral chest wall for the last two years. According to the patient, the lesion started as few asymptomatic erythematous papules that coalesced to form plaque in the lateral part of left chest. This plaque increased gradually leaving a healed atrophic center. The borders were covered with thick crusts. There was no history of cough, fever, night sweats, or weight loss. There was no history of tuberculosis or malignancy in other family members. Patient was nonsmoker and non-alcoholic beverage drinker.

**Figure 1.**
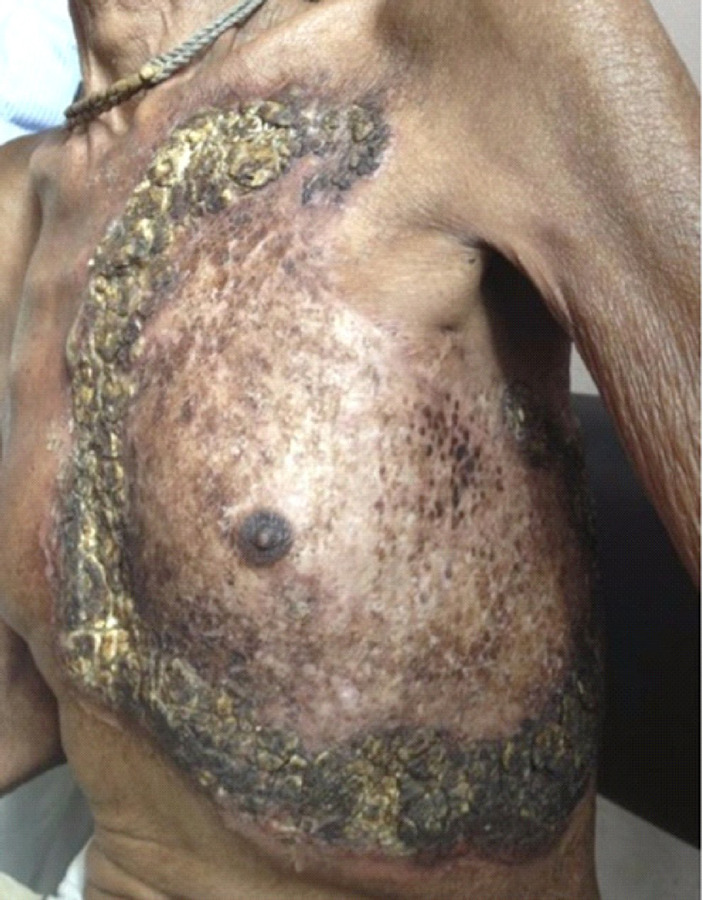
Clinical photograph showing a single large plaque on the whole of left chest.

On examination, a single large plaque on left anterior chest wall covering the whole of the left chest superiorly from clavicular line involving the entire left chest up to the subcostal region and extending posteriorly to infrascapular region, sparing the left axilla. The approximate size of the plaque was 30 × 30 cm^2^ (Figure 1). It was arcuate in shape with clearing of the adjacent areas with few areas of atrophy. The parasternal and subcostal margin of the plaque had thick adherent crusts on an erythematous base. There were areas of scarring and atrophy in the lesion. The plaque was non-tender and diascopy was negative for apply jelly nodules. The corresponding lymph nodes were not enlarged.

Complete blood counts were within normal limits but a raised erythrocyte sedimentation rate of 105mm/hr was noted. Mantoux test performed showed an induration of 20 mm. Chest x-ray showed no abnormalities. Sputum for acid-fast bacilli did not show any acid-fast bacilli. Liver function test and renal function test were all within normal limits.

An incisional biopsy done from the plaque showed pseudo-epitheliomatous hyperplasia, neutrophilic abscess formation in the corneal layer. Underlying dermis showed ill-defined epitheloid cell granuloma, langhans giant cells and a surrounding inflammatory infiltrates consisting of plasma cells, lymphocytes and neutrophilic micro abscesses and fibrosis (igure 2a and 2b).

**Figure 2a.**
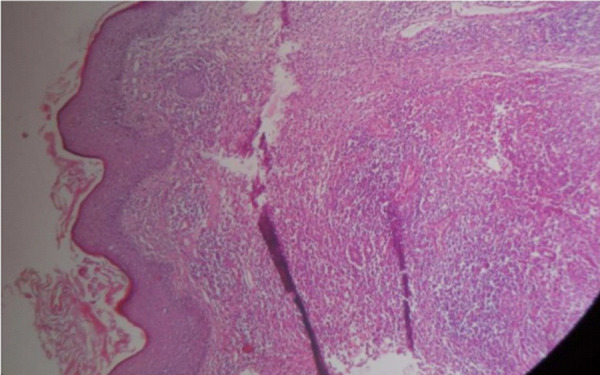
HPE shows pseudo-epitheliomatous hyperplasia, neutrophilic abscess formation in the corneal layer. Underlying dermis shows ill-defined epitheloid cell granuloma (40× magnification, H & E stain).

**Figure 2b.**
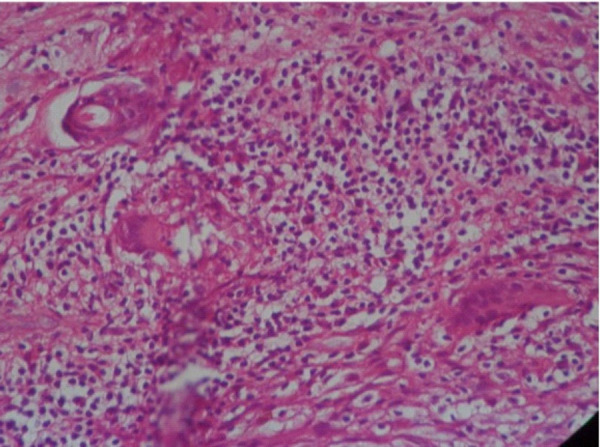
HPE showing epitheloid cell granuloma, Langhans giant cells, surrounding mixed inflammatory infiltrate consisting of plasma cells and lymphocytes (100× magnifications, H & E stain).

The patient was diagnosed as a case of lupus vulgaris and was started on anti-tubercular treatment category 1 and was advised for follow-up.

## DISCUSSION

Cutaneous tuberculosis results from a chronic infection by Mycobacterium tuberculosis, M. bovis and occasionally by the Calmette-Guerin bacillus. The clinical manifestations are variable which depends on the interaction of several factors including the site of infection and the host's immunity.^2^

Lupus vulgarisis a chronic and progressive form of cutaneous tuberculosis. Lesions are generally solitary and found on the head and neck region. The clinical presentation can be of several different clinical appearances.^3^ It can be transmitted either through hematogenous or lymphatic spread but most commonly through contiguous extension. Rarely, it can also occur from exogenous inoculation or on post- BCG scar.^4^ The common locations of lupus vulgaris are the lower half of the body involving legs, thighs, buttocks, and feet.^5^ The different variants of lupus vulgaris includes plaque, ulcerative, mutilating, vegetative, papular or nodular and hypertrophic form. A prominent feature of lupus vulgaris is atrophic scarring, with or without ulceration.^6^ Typically, the histopathology shows tuberculoid granulomas composed of lymphocytes, plasma cells, epitheloidand giant cells, scant or absent central caseation, in the superficial dermis. The epidermis is usually hyperplastic, but may be atrophic or ulcerated. Stain for acid fast bacilli is usually negative. A characteristic feature of lupus vulgaris is its extremely chronic course. In this reported case, the patient had a large plaque within a short period of just over 2 years. The largest reported case of lupus vulgaris in literature is from India with size of 60×45 cm and 40× 50cm.^7^

## Consent:

**JNMA Case Report Consent Form**was signed by the patient and the original is attached with the patient's chart.

## Conflict of Interest:

**None.**
